# Parental Acceptance of Passive Protective Stabilization During Pulp Therapy in Primary and Young Permanent Dentition: A Systematic Review and Meta-Analysis

**DOI:** 10.3390/jcm15062200

**Published:** 2026-03-13

**Authors:** Carolina Caleza-Jiménez, Cira Suárez-Marchena, Lucy Chandler-Gutiérrez, Juan J. Segura-Egea, Carmen Machuca-Portillo

**Affiliations:** 1 Pediatric Dentistry Division, Department of Stomatology, School of Dentistry, University of Sevilla, C/Avicena s/n, 41009 Sevilla, Spain; csuarez1@us.es (C.S.-M.); chandler@us.es (L.C.-G.); 2 Endodontic Division, Department of Stomatology, School of Dentistry, University of Sevilla, C/Avicena s/n, 41009 Sevilla, Spain; segurajj@us.es

**Keywords:** behavior management, dental anxiety, passive protective stabilization, pediatric dentistry, pulp therapy

## Abstract

**Background:** Passive protective stabilization (PPS) remains controversial in pediatric dentistry, particularly in invasive procedures such as pulp therapy. This systematic review and meta-analysis aimed to evaluate current parental acceptance of PPS. **Methods:** A systematic search of PubMed/MEDLINE, Cochrane Library, Scopus, and Embase was conducted up to 25 November 2025. Cross-sectional studies assessing parental acceptance of PPS in children aged 2–10 years were included. Studies published in English within the last 10 years and including at least 100 participants were eligible. Case reports, reviews, editorials, and studies not aligned with the objectives were excluded. Risk of bias was assessed using the Joanna Briggs Institute checklist. A random-effects meta-analysis with logit transformation was performed. Heterogeneity was quantified using I^2^ statistics. Certainty of evidence was evaluated using GRADE. **Results**: Five cross-sectional studies, including 1005 parents, were included. The pooled parental acceptance of PPS was 48.9% (95% CI: 29.0–69.2%), with substantial heterogeneity (I^2^ = 97%). Acceptance was consistently higher in emergency situations and among parents of children with special health care needs. Three studies showed low risk of bias and two moderate risk. The overall certainty of evidence was rated as very low due to inconsistency and imprecision. **Conclusions:** Parental acceptance of PPS is context-dependent and influenced by treatment urgency and patient characteristics. Given the high heterogeneity and low certainty of evidence, results should be interpreted cautiously. Further high-quality research is required.

## 1. Introduction

Children’s behavior in the dental setting is closely related to their age and level of cognitive and emotional development, which may influence their ability to cope with stressful or anxiety-provoking situations, such as pulpal treatment. Despite significant advances in pulpal therapies, particularly with the introduction of bioactive materials, the management of these lesions according to the severity of pulpal inflammation and the child’s behavior may still be challenging [1]. Difficulties in managing uncooperative or disruptive behavior—often resulting in treatment delays or even the inability to complete dental procedures—are more frequently observed in children with dental fear and anxiety [2].

In addition to the developmental immaturity of pediatric patients, pulpal treatments typically require longer chair time, which further increases behavioral management challenges. Moreover, the prevalence of molar–incisor hypomineralization (MIH) has increased in recent years [3]. In severe cases, MIH is characterized by rapidly progressing coronal breakdown and pulpal involvement, often accompanied by a history of dental hypersensitivity. These children may be reluctant to undergo even a basic dental examination. Severely affected molars frequently present extensive structural disintegration and require complex restorative procedures [2]. Consequently, children with MIH tend to receive more dental treatment than unaffected peers [3].

Regardless of the origin of dental fear or anxiety, an appropriate clinical approach is essential when managing pediatric patients requiring invasive procedures. Shorter, well-structured appointments that promote a calm environment in the dental chair may facilitate improved cooperation and enhance the successful completion of pulpal treatment. Currently, a wide range of behavior management techniques is available to support child cooperation during dental care. All these approaches aim to establish effective communication to reduce fear and anxiety, build a trusting relationship with the child, and enable the dentist to provide high-quality treatment while fostering a positive attitude toward oral health and future dental care [4,5,6].

According to the American Academy of Pediatric Dentistry (AAPD), behavior management techniques are classified as either basic or advanced [7]. Basic techniques include communication strategies and behavior modification. However, a subset of children cannot be managed using basic techniques alone and may require advanced approaches, such as protective stabilization, sedation, or general anesthesia [8]. Protective stabilization may be classified as active or passive. Active restraint involves immobilization by another person, such as a parent or dental professional, whereas passive restraint refers to the use of a mechanical or physical device, such as a Papoose Board^®^ (Olympic Medical Corporation, Seattle, WA, USA) or Pedi-Wrap^®^ (The Medi-Kid Co., Hemet, CA, USA) [7].

Protective stabilization is considered an advanced behavior management technique in contemporary pediatric dentistry. Recommendations regarding its use were developed by the AAPD Clinical Affairs Council, initially adopted in 2013 [9] and most recently revised in 2025 [10]. When appropriately applied, protective stabilization can limit uncontrolled movements, reduce the risk of injury to the patient and dental staff, and facilitate the delivery of safe and effective dental care. Nevertheless, if used incorrectly or without adequate communication and informed consent, protective stabilization may be perceived as coercive or harmful, raising ethical concerns [10].

The decision to employ passive protective stabilization (PPS) should be based on multiple factors, including the child’s oral health needs, emotional and cognitive development, medical and physical conditions, and the potential impact on both treatment quality and patient well-being [11]. Children with special health care needs (SHCN), in particular, may require protective stabilization to enable the completion of necessary dental procedures [12]. Aggressive, impulsive, or involuntary movements during treatment may place both the child and dental personnel at risk of injury [13]. Several studies have demonstrated that sensory-adapted environments and techniques—such as the deep-pressure effect produced by immobilization devices—may provide comfort and reduce stress without causing harm in children with special needs receiving medical or dental care [13,14]. Importantly, long-term dental anxiety or negative memories have not been consistently reported among children treated with these systems [15].

Despite these potential benefits, the use of PPS in pediatric dentistry remains controversial, largely due to ethical concerns regarding its possible psychological impact. Contemporary parenting trends, greater sensitivity to children’s emotional responses, and evolving societal expectations regarding discomfort during healthcare procedures may also influence parental perceptions of restraint techniques [15,16].

Previous studies have examined factors affecting parental acceptance of PPS, including socioeconomic status, educational level, race, and cultural background. However, few recent investigations have focused specifically on parental acceptance of PPS in the context of necessary and invasive treatments, such as pulp therapy. Improved understanding of parental attitudes toward this technique may enhance communication between dental professionals and caregivers and contribute to better clinical decision-making in pediatric dentistry.

Invasive dental procedures such as pulp therapy represent a significant clinical burden in pediatric dentistry. The global prevalence of untreated dental caries in primary dentition has been estimated at approximately 46%, frequently progressing to pulpal involvement requiring urgent intervention. Moreover, dental anxiety affects between 10% and 20% of children worldwide, contributing to behavioral management challenges and delayed treatment. Although pharmacological techniques such as sedation and general anesthesia have been increasingly utilized, their availability is often limited by economic, logistical, and safety considerations. Recent systematic reviews suggest a growing demand for pharmacological management; however, high-quality comparative evidence regarding long-term psychological outcomes remains limited. Within this context, understanding parental acceptance of non-pharmacological advanced techniques such as PPS is of clinical and ethical relevance.

Given the limited and heterogeneous nature of the available evidence, as well as the ongoing debate regarding the indications, risks, and acceptability of PPS, this systematic review was conducted to assess current parental acceptance of this technique in invasive dental procedures, particularly pulp therapy in primary and young permanent dentition.

## 2. Materials and Methods

### 2.1. Protocol and Registration

This systematic review was conducted in accordance with the Preferred Reporting Items for Systematic Reviews and Meta-Analyses (PRISMA) 2020 guidelines [17]. The review protocol was prospectively registered in the International Prospective Register of Systematic Reviews (PROSPERO) under the registration number CRD420251251666. The protocol is available at: https://www.crd.york.ac.uk/PROSPERO/view/CRD420251251666 (accessed on 13 December 2025). Minor differences in authorship between the PROSPERO registration and the final submitted manuscript reflect changes in academic contributions during manuscript preparation. No modifications were made to the registered research question, eligibility criteria, or methodological approach.

### 2.2. Review Question

The primary research question of this systematic review was: “What is the level of parental acceptance of PPS during invasive dental procedures?” This question was developed using the PICOS framework [18] as follows:

Population: Parents of children aged 2 to 10 years. Intervention: Completion of a questionnaire assessing parental acceptance of PPS during their child’s dental treatment.

Comparators: Differences in parental acceptance according to the type of dental treatment (routine vs. emergency).

Outcomes: Degree of parental acceptance of PPS.

Study design: Cross-sectional studies.

### 2.3. Eligibility Criteria

Eligibility criteria were established according to the Strength of Recommendation Taxonomy (SORT) guidelines.

Inclusion criteria.

Studies meeting all the following inclusion criteria were considered eligible: studies published within the last 10 years, evaluating parental acceptance of PPS during pediatric dental treatment, published in English, and including a minimum sample size of 100 parents. In addition, studies were required to clearly describe the clinical context in which PPS was evaluated (routine vs. emergency care) and to report quantitative data enabling calculation of parental acceptance proportions. Studies evaluating mixed behavior management techniques were included only if PPS-specific data could be extracted separately.

Exclusion criteria: case series, reviews, letters, editorials, and commentaries were excluded, as well as studies not aligned with the objectives of the present review.

### 2.4. Search Strategy

A comprehensive literature search was performed in four electronic databases: PubMed/MEDLINE, Cochrane Library, Scopus, and Embase. The search strategy combined controlled vocabulary (MeSH and Emtree terms where applicable) and free-text keywords related to “behavior management,” “protective stabilization,” “parents,” “pediatric dentistry,” “dental pulp therapy,” and “dental anxiety.” Boolean operators (“AND” and “OR”) were used to optimize sensitivity and specificity. The search strategy was adapted for each database according to its specific indexing system. Complete search strategy in PubMed/MEDLINE is shown in Supplementary Material S2. The last search was conducted on 25 November 2025. In addition, reference lists of all included studies were manually screened to identify potentially relevant articles. No additional studies meeting the predefined inclusion criteria were identified through the expanded database search.

### 2.5. Selection of Studies

Three authors individually selected the articles (C.C.-J., L.C.-G., and C.M.-P.). A manual search of the reference lists of the included articles was conducted. These three authors examined both the title and abstract of the retrieved articles, determined their eligibility, and analyzed the full text of the studies that initially appeared to meet the inclusion criteria. Disagreements regarding eligibility were resolved through discussion and consensus. Subsequently, the full texts of all selected studies were obtained. Finally, a manual search was conducted to identify additional relevant studies. Discrepancies among the reviewers were resolved through discussion until a consensus was reached.

### 2.6. Data Extraction

Two authors (C.S.-M. and J.J.S.-E.) extracted the data, and three reviewers (C.C.-J., L.C.-G., and C.M.-P.) verified the tabulated data to ensure the absence of typographical errors and performed the analysis of the articles. Articles with discrepancies were analyzed. To synthesize the data, some details were extracted from the studies: author and year of publication, study design, sample size, age, sex, and results. The pooled estimates from the studies were analyzed using a random-effects meta-analysis.

### 2.7. Data Synthesis and Analysis

The primary outcome analyzed was the proportion of parents accepting passive protective stabilization (PPS). A quantitative synthesis was performed to estimate the overall parental acceptance across included studies.

For each study, the proportion of parental acceptance and corresponding 95% confidence intervals (CIs) were calculated. Meta-analysis was conducted using Review Manager (RevMan), version 5.4 (The Cochrane Collaboration, Copenhagen, Denmark). Given the anticipated clinical and methodological heterogeneity among studies—particularly regarding cultural context, urgency of treatment (routine vs. emergency), and inclusion of children with special health care needs—a random-effects model (DerSimonian and Laird method) was applied to account for both within-study and between-study variability.

Pooled estimates were calculated using inverse variance weighting. Statistical heterogeneity was assessed using the Chi-square (Cochran Q) test, with statistical significance set at *p* < 0.10 due to the low power of the test in meta-analyses with few studies. The magnitude of heterogeneity was quantified using the I^2^ statistic and interpreted according to Cochrane Handbook recommendations as follows: 0–40% might not be important, 30–60% may represent moderate heterogeneity, 50–90% may represent substantial heterogeneity, and 75–100% considerable heterogeneity.

Given the high level of heterogeneity observed, the pooled estimate was interpreted as a descriptive summary rather than a definitive or generalizable measure of parental acceptance. Forest plots were generated using RevMan 5.4 to visually present individual study estimates and the pooled effect size. All analyses were conducted using a significance level of *p* < 0.05, except for heterogeneity testing as specified above.

### 2.8. Risk of Bias Assessment

The methodological quality of the included analytical cross-sectional studies was assessed using the Joanna Briggs Institute (JBI) Critical Appraisal Tool for Analytical Cross-Sectional Studies [19]. This instrument comprises eight domains evaluating the clarity of inclusion criteria, detailed description of study participants and settings, validity and reliability of exposure measurement, use of objective and standard criteria for outcome assessment, identification and management of potential confounding factors, validity and reliability of outcome measurement, and appropriateness of the statistical analysis. Each item was rated as “Yes” or “No,” and an overall risk of bias was classified as low or moderate based on the number of domains adequately fulfilled. Two independent reviewers evaluated each study, and disagreements were resolved by consensus.

### 2.9. Analysis of GRADE Evidence Levels

The certainty of evidence for the main outcome was evaluated using the GRADE (Grading of Recommendations Assessment, Development and Evaluation) approach [20]. This assessment considered five domains: (1) risk of bias, (2) inconsistency, (3) indirectness, (4) imprecision, and (5) publication bias. Three reviewers (C.S.-M., C.M.-P. and J.J.S.-E) independently assessed each domain, and any discrepancies were resolved through discussion and consensus.

## 3. Results

### 3.1. Study Selection

**Figure 1:**
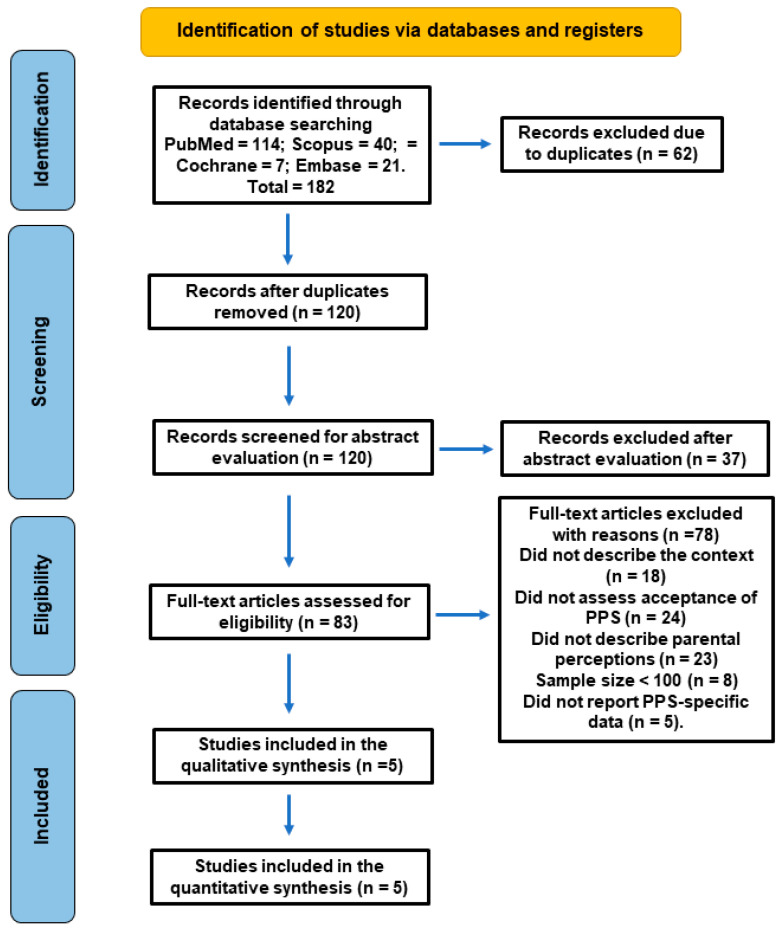
PRISMA flow diagram illustrating the study selection and screening process.

The study selection process is illustrated in Figure 1. The initial database search identified a total of 182 records. After removal of duplicates (*n* = 62 excluded) and the screening for title and abstract evaluation (*n* = 37 excluded), 83 articles were selected for further evaluation. After full-text reading, 78 studies were excluded for different reasons (Table S1): did not describe the context (routine vs. emergency care) (*n* = 18), did not assess acceptance of PPS (*n* = 24), did not describe parental perceptions (*n* = 23), sample size < 100 (*n* = 8), or did not report PPS-specific data (*n* = 5). Finally, five studies fulfilled all eligibility criteria and were included in the final qualitative synthesis [21,22,23,24,25].

### 3.2. Characteristics of the Included Studies

The main characteristics of the included studies are summarized in Table 1. The five studies were published between 2016 [25] and 2024 [21] and were conducted in different geographical settings, including Europe [21,22,23,24] and the United States [25]. Sample sizes ranged from 105 [25] to 440 [21] parents. Across the included studies, parental age was inconsistently reported. Mean age was available in only two studies, ranging from 38.5 [22] to 39.3 [25] years, while the remaining studies did not report this variable. In contrast, the proportion of mothers among participants was consistently high in all studies, varying from 69.9% [24] to 87.1% [22]. This predominance of mothers indicates that maternal perspectives largely informed the reported acceptance of behavior management techniques.

The overall parental acceptance of PPS varied considerably among the included studies, ranging from 22.85% [23] to 64.05% [22]. Two studies reported acceptance rates exceeding 55%, both of which were conducted among parents of children with special health care needs (SHCN) [21,22].

Across all included studies, parental acceptance of passive restraint was consistently higher in emergency or urgent treatment situations compared with routine dental care. These differences were statistically significant in all studies that performed comparative analyses. In addition, children with SHCN were associated with higher parental acceptance of advanced behavior management techniques, including passive restraint, when compared with neurotypical children.

### 3.3. Data Analysis and Quantitative Synthesis

A total of 1005 parents were analyzed in the five included articles [21,22,23,24,25], of which 490 accepted PPS.

**Figure 2:**
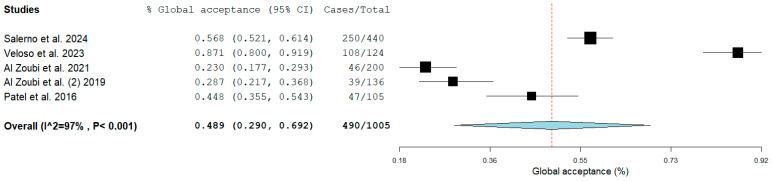
Forest plot of the pooled global parental acceptance of PPS. Individual study proportions with 95% confidence intervals are shown, together with the overall pooled estimate calculated using a random-effects meta-analysis with logit transformation. Substantial heterogeneity was observed among the included studies (I^2^ = 97%, *p* < 0.001) [21,22,23,24,25].

The meta-analysis of five cross-sectional studies, including a total of 1005 participants, showed a pooled global acceptance rate, calculated using a random-effects meta-analysis with logit transformation, of 48.9% (95% CI: 29.0–69.2%). Individual study estimates varied widely, ranging from 23.0% [23] to 87.1% [22], reflecting substantial variability across studies. The heterogeneity was very high (I^2^ = 97%, *p* < 0.001), indicating considerable between-study differences that may be related to variations in clinical scenarios, urgency of treatment, parental characteristics, and types of behavior management techniques evaluated (Figure 2).

Given the magnitude of heterogeneity, the pooled estimate should be interpreted as a descriptive summary rather than a precise measure of effect. The variability observed across studies likely reflects differences in clinical context, particularly treatment urgency and the inclusion of children with special health care needs.

### 3.4. Risk of Bias Assessment

The risk of bias of the included studies was assessed using the Joanna Briggs Institute Critical Appraisal Checklist for Analytical Cross-Sectional Studies. The detailed assessment is presented in Table 2.

Three studies were judged to have a low risk of bias [21,23,24], while two studies presented a moderate risk of bias [22,25]. The most common sources of potential bias were related to the absence of objective criteria for outcome measurement and insufficient identification or management of confounding factors. Overall, the methodological quality of the included studies was considered acceptable for qualitative synthesis.

### 3.5. GRADE Assessment of the Certainty of Evidence

**Figure 3:**
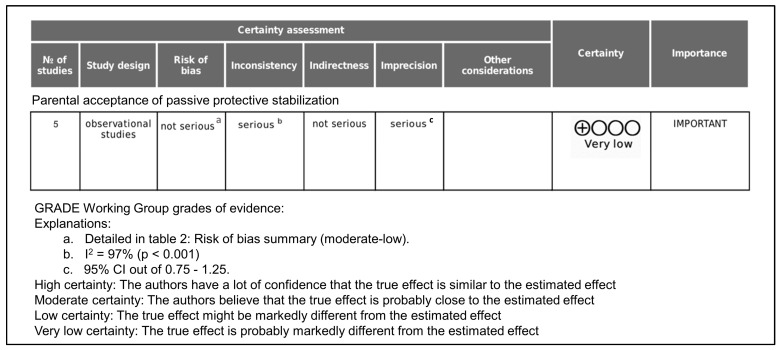
Grade assessment of evidence certainty.

The certainty of evidence regarding parental acceptance of PPS was assessed using the GRADE approach, considering five domains: risk of bias, inconsistency, indirectness, imprecision, and publication bias (Figure 3).

The overall risk of bias was considered not serious. As shown in Table 2, three of the five included studies were assessed as having a low risk of bias, while two studies presented a moderate risk of bias, mainly due to limitations in confounding factor identification and outcome measurement. Given that no studies were judged to have a high risk of bias, the certainty of evidence was not downgraded for this domain.

A serious level of inconsistency was identified. The meta-analysis demonstrated substantial statistical heterogeneity (I^2^ = 97%), indicating marked variability in parental acceptance estimates across studies. This heterogeneity likely reflects differences in study populations, clinical contexts (routine vs. emergency care), and inclusion of children with special health care needs. Therefore, the certainty of evidence was downgraded by one level for inconsistency.

The evidence was considered not serious for indirectness. All included studies directly addressed the population of interest (parents of pediatric dental patients), the intervention (PPS), and the outcome (parental acceptance). Although some studies included broader advanced behavior management techniques, acceptance of PPS was explicitly evaluated in all cases. Consequently, no downgrade was applied for indirectness.

The certainty of evidence was downgraded by one level for imprecision. The pooled estimate showed a wide 95% confidence interval (0.290–0.692), reflecting uncertainty in the magnitude of parental acceptance. In addition, the relatively small number of included studies limited the precision of the overall estimate.

Publication bias was considered unclear but not suspected. The limited number of included studies (<10) precluded formal assessment using funnel plots or statistical tests. No evidence of selective outcome reporting was identified; therefore, the certainty of evidence was not downgraded for this domain.

Considering the above domains, the overall certainty of evidence for parental acceptance of PPS was rated as very low.

## 4. Discussion

The objective of this systematic review and meta-analysis was to assess current parental acceptance of this technique in invasive dental procedures, particularly pulp therapy in primary and young permanent dentition. Overall, the findings of the systematic review demonstrate substantial variability in parental acceptance of PPS.

Nevertheless, two consistent patterns emerged across all included studies. On the one hand, higher parental acceptance of passive restraint in emergency situations, particularly when dental pain or urgent intervention was required. On the other hand, greater acceptance among parents of children with special health care needs, compared with parents of neurotypical children. These trends were observed regardless of geographic location, study design, or sample characteristics. However, according to the GRADE approach, the certainty of evidence was rated as very low, mainly due to substantial heterogeneity and imprecision.

In recent years, there has been a growing tendency among parents to request sedation or general anesthesia for pediatric dental procedures, particularly for invasive treatments such as pulp therapy. This trend may be driven by the desire to minimize children’s anxiety, discomfort, and distress during dental care, as well as to prevent potentially negative dental experiences [26,27]. However, increasing concerns regarding the safety of pharmacological behavior management techniques—especially in children with underlying medical conditions—and economic limitations affecting access to these treatments have renewed interest in alternative approaches. Within this context, PPS has re-emerged as a potential behavior management option for selected pediatric patients [28]. The present systematic review explored current parental acceptance of PPS in pediatric dental procedures, with particular emphasis on invasive and urgent treatments.

The findings of this review indicate considerable variability in overall parental acceptance of PR, with reported acceptance rates ranging from low to moderate across studies. Importantly, no clear temporal trend toward increasing or decreasing acceptance was observed over the past decade. Nevertheless, two consistent patterns emerged. First, parental acceptance of passive restraint was significantly higher in emergency situations requiring urgent dental intervention. Second, parents of children with SHCN demonstrated greater acceptance of PPS compared with parents of neurotypical children.

The quantitative synthesis conducted in this review provides an exploratory overview of global parental acceptance of PPS. Although a pooled acceptance estimate was calculated using a random-effects model with logit transformation, a very high level of heterogeneity was observed across studies. This finding underscores the context-dependent nature of parental acceptance and reflects substantial differences in study populations, clinical scenarios, and outcome assessment methods. Therefore, the pooled estimate should not be interpreted as a uniform or generalizable acceptance rate, but rather as a descriptive summary of highly variable evidence. Importantly, the observed heterogeneity highlights the influence of treatment urgency and patient-related factors, reinforcing the need for individualized clinical decision-making when considering PPS.

Children with SHCN are defined as those who have, or are at increased risk of developing, chronic physical, developmental, behavioral, or emotional conditions that require health services beyond those needed by the general pediatric population [29]. Dental treatment in this group is often complicated by limited cooperation, heightened sensory sensitivity, involuntary movements, or communication difficulties, which may increase the risk of injury and compromise treatment outcomes. Several studies have reported that immobilization devices providing deep-pressure sensory input may have a calming effect in children with SHCN, including those with autism spectrum disorder (ASD), without inducing physiological stress or long-term psychological harm [13,30,31]. These findings may help explain the higher level of parental acceptance of passive restraint observed in studies involving children with SHCN, as caregivers in this population are often familiar with structured behavioral and therapeutic interventions in other healthcare settings.

Families of children with SHCN also face substantial barriers to accessing dental care, including limited availability of specialized services and increased treatment complexity. In such circumstances, parents may prioritize the completion of necessary dental treatment over concerns related to temporary discomfort or restraint. Moreover, some parents may perceive passive restraint as a preferable alternative to general anesthesia, particularly given concerns about systemic risks, recovery time, and costs associated with pharmacological approaches [28,32,33]. This perspective is consistent with the higher acceptance rates reported in studies involving children with SHCN included in the present review [21,22].

Another important finding of this review is the influence of treatment urgency on parental acceptance of PPS. Across all included studies, acceptance was significantly higher in emergency settings compared with routine dental care. Pulpal pathology in children, commonly resulting from deep caries or dental trauma, is frequently associated with acute pain that may be spontaneous or nocturnal, necessitating prompt intervention. In these situations, parents may be more willing to accept advanced behavior management techniques if they facilitate immediate pain relief and prevent treatment delays or tooth loss. These findings align with earlier studies demonstrating that parental attitudes toward behavior management techniques are strongly influenced by the perceived necessity and urgency of treatment [34,35,36,37].

Age and developmental stage may also play a role in parental acceptance of passive restraint. Younger children often exhibit reduced ability to understand dental procedures and follow instructions, which may contribute to higher levels of uncooperative behavior during invasive treatments. Previous studies have shown that parental acceptance of restraint techniques tends to be higher among parents of younger children, likely reflecting pragmatic considerations related to treatment feasibility [38,39]. Invasive procedures such as pulp therapy involve multiple potentially distressing stimuli, including local anesthesia, unfamiliar instruments, dental noise, and prolonged chair time, all of which may exacerbate anxiety and negatively affect cooperation [40].

Parental characteristics, including anxiety levels and personality traits, have also been shown to influence acceptance of behavior management techniques. Several studies have reported that parents with higher dental anxiety or negative personality traits are less likely to accept PPS [38,41,42]. Conversely, other research suggests that parents experiencing moderate to high anxiety may prefer passive restraint when faced with challenging child behavior, particularly if restraint is perceived as facilitating treatment completion and reducing procedural risk [43]. These apparently conflicting findings highlight the complexity of parental decision-making and underscore the importance of individualized communication strategies tailored to parental concerns and expectations.

Effective communication between dental professionals and parents plays a central role in shaping acceptance of passive restraint. Clear explanations regarding the purpose, benefits, and limitations of protective stabilization, as well as a discussion of available alternatives, are essential. The use of visual aids—such as videos, photographs, or real-time observation—has been shown to improve parental understanding and acceptance of restraint techniques [30,44,45,46]. Importantly, PPS should be presented as a protective and supportive measure rather than as a punitive intervention. Creating a collaborative decision-making environment in which parents are encouraged to express concerns and actively participate in treatment planning may further enhance acceptance and trust.

Despite the clinical relevance of these findings, this review has several limitations. This systematic review and meta-analysis presents several important limitations that must be considered when interpreting the findings.

First, all included studies were cross-sectional in design. Consequently, causal inferences cannot be drawn, and the results reflect associations rather than determinants of parental acceptance. Cross-sectional surveys are also inherently susceptible to response bias and may not accurately capture real-time decision-making in stressful clinical situations.

Second, although the search strategy was expanded to four major electronic databases (PubMed/MEDLINE, Cochrane Library, Scopus, and Embase) and complemented by manual reference screening, the possibility of missing relevant studies cannot be completely excluded. Nevertheless, the inclusion of these additional databases substantially reduces the likelihood of publication omission and strengthens the comprehensiveness of the search. The restriction to English-language publications may also have introduced language bias.

Third, the number of eligible studies was limited, and the total number of included investigations (*n* = 5) restricts the robustness of quantitative synthesis. Meta-analyses based on a small number of studies are more vulnerable to instability in pooled estimates and limited capacity for formal assessment of publication bias. Funnel plot asymmetry and statistical tests for small-study effects could not be reliably performed.

Fourth, substantial statistical heterogeneity was observed (I^2^ = 97%), indicating considerable variability across studies. This heterogeneity likely reflects differences in geographic and cultural contexts, healthcare systems, parental expectations, study populations, inclusion of children with special health care needs, urgency of treatment scenarios, and measurement instruments. Such heterogeneity limits the external validity and generalizability of the pooled estimate. Although a random-effects model was appropriately applied to account for between-study variability, the summary proportion should be interpreted as descriptive rather than definitive.

Fifth, parental acceptance was measured using non-standardized instruments across studies. Variability in questionnaire design, Likert scale structure, dichotomization methods, and contextual framing of PPS (routine vs. emergency scenarios) may have influenced reported acceptance rates. Moreover, some studies assessed hypothetical acceptance rather than acceptance following actual clinical experience, which may not accurately reflect real-world parental decision-making. Social desirability bias may also have affected responses, particularly when questionnaires were administered in clinical environments.

Sixth, confounding variables were inconsistently identified and controlled across studies. Factors such as parental dental anxiety, socioeconomic status, cultural beliefs, prior dental experiences, and child behavioral characteristics may substantially influence acceptance but were not uniformly adjusted for. This limitation further contributes to uncertainty in pooled estimates.

Seventh, subgroup analyses (e.g., emergency vs. routine care, children with special health care needs vs. neurotypical children) could not be formally meta-analyzed due to insufficient homogeneous data. As a result, observed patterns were derived from qualitative synthesis rather than statistically robust subgroup comparisons.

Eighth, the statistical synthesis itself presents inherent limitations. Meta-analysis of proportions may be sensitive to extreme values and distributional assumptions. Although appropriate methods were applied and heterogeneity was quantified according to Cochrane recommendations, the wide confidence intervals observed indicate imprecision in the pooled estimate.

Finally, the certainty of evidence was rated as very low using the GRADE framework, primarily due to serious inconsistency and imprecision. This rating reflects not only statistical heterogeneity but also methodological variability and limited study number. Therefore, conclusions should be interpreted with caution and should not be considered definitive clinical guidance.

Future research should prioritize longitudinal and prospective designs, standardized outcome measures, consistent reporting of contextual variables, and larger multicenter samples to improve precision and generalizability. Harmonization of measurement instruments and clearer operational definitions of PPS would substantially strengthen the evidence base.

## 5. Conclusions

This systematic review suggests that parental acceptance of passive protective stabilization (PPS) in pediatric dentistry is highly context-dependent and influenced primarily by treatment urgency and the presence of special health care needs. Acceptance appears to increase in emergency situations requiring immediate intervention and among parents of children with special health care needs.

However, given the substantial heterogeneity among studies and the very low certainty of evidence according to the GRADE framework, these findings should be interpreted with caution. The pooled estimate represents a descriptive summary of variable evidence rather than a definitive measure of parental acceptance.

Within these limitations, PPS may be considered a potential adjunctive behavior management technique in carefully selected cases, particularly when basic behavior guidance strategies are insufficient and when pharmacological approaches are contraindicated, unavailable, or declined. Its use should always be guided by individualized clinical judgment, ethical considerations, and transparent communication with caregivers.

Future well-designed studies using standardized outcome measures and prospective methodologies are needed to clarify parental perceptions and to strengthen the evidence base regarding the appropriate and ethical use of PPS in pediatric dental practice.

## Figures and Tables

**Table 1:** Characteristics of the included studies.

Author, Year and Country	Sample	Mean Age (y)	Sex (%)	Global Acceptance (%)	Results
Salerno et al. 2024, Italy [21]	440 parents	Not reported	77.6 mothers	56.8	Parents of children with special needs showed a higher acceptance rate of advanced BMTs compared to parents of neurotypical children. Differences between the two groups of parents in acceptance of active restraint in emergency settings, passive restraint in routine settings, and Deep sedation/general anesthesia in both settings were observed (*p* < 0.01).
Veloso et al. (2023), Spain [22]	124 parents	38.5	87.1 mothers	64.1	PR (*p* = 0.029) was significantly more accepted when used in emergencies than during routine consultations.
Al Zouby et al. (2021), Germany [23]	200 parents	Not reported	74.0 mothers	22.9	The acceptance of passive restraint increased significantly in both groups of parents (Germany vs. Jordan) when the treatment was urgent.
Al Zouby et al. (2019), Germany [24]	136 parents	Not reported	69.9 mothers	28.9	The acceptance of passive restraint was significantly higher when the treatment was urgent (*p* < 0.001, paired sample *t* test).
Patel et al. (2016), USA [25]	105 parents	39.3	81.0 mothers	44.4	As urgency, convenience, and previous experience increased, parental acceptance of the technique increased. As cost of treatment increased, parental acceptance decreased. Ratings between the children’s hospital group and private practice group differed, as did the demographic variables of insurance, income, and race.

**Table 2:** Risk of bias assessment.

Study	Were the Criteria for Inclusion in the Sample Clearly Defined?	Were the Study Subjects and the Setting Described in Detail?	Was the Exposure Measured in a Valid and Reliable Way?	Were Objective, Standard Criteria Used for Measurement of the Condition?	Were Confounding Factors Identified?	Were Strategies to Deal with Confounding Factors Stated?	Were the Outcomes Measured in a Valid and Reliable Way?	Was Appropriate Statistical Analysis Used?	ROB
Salerno et al. [21]	Yes	Yes	Yes	Yes	Yes	Yes	Yes	Yes	Low
Veloso et al. [22]	Yes	Yes	Yes	No	No	Yes	Yes	Yes	Moderate
Al Zouby et al. [23]	Yes	No	Yes	Yes	Yes	Yes	Yes	Yes	Low
Al Zouby et al. [24]	Yes	Yes	Yes	Yes	Yes	Yes	Yes	Yes	Low
Patel et al. [25]	Yes	Yes	Yes	No	No	Yes	Yes	Yes	Moderate

## Data Availability

No new data were created or analyzed in this study.

## Supplementary Materials

The following supporting information can be downloaded at: https://www.mdpi.com/article/10.3390/jcm15062200/s1, Table S1. Excluded studies and their reasons for exclusion. Supplementary Material S2: Full PubMed Search Strategy. PRISMA 2020 Checklist.
