# Supplementary material for: Parental Acceptance of Passive Protective Stabilization During Pulp Therapy in Primary and Young Permanent Dentition: A Systematic Review and Meta-Analysis
Source: J Clin Med. 2026 Mar 13;15(6):2200. doi: 10.3390/jcm15062200 (PMC13026584; doi:10.3390/jcm15062200)
Supplement: Supplementary file 1 [file jcm-15-02200-s001.zip › jcm-4161264-supplementary.pdf]

Supplementary material Table S1. Excluded studies and their reasons for exclusion.

Reasons	Excluded Studies
Did not describe the context (routine vs. emergency care)	Aldhelai et al. 2023<br>Candan et al. 2023<br>Chang et al. 2018<br>Chhabra et al. 2021<br>da Silva et al. 2021<br>Desai et al. 2019<br>Faghihian et al. 2023<br>Gomez et al. 2024<br>Guinot et al. 2021<br>Gupta and Khajuria 2019<br>Hasheminejad et al. 2025<br>Manopetchkasem et al. 2023<br>Martinez et al. 2019<br>Sabbagh et al. 2021<br>Sankpal et al. 2025<br>Seangpadsa et al. 2020<br>Theriot et al. 2019<br>Wilson et al. 2016
Did not assess acceptance of PPS	Acharya et al. 2024<br>Aguirre et al. 2020<br>Albaker et al. 2025<br>Alrumaim et al. 2025<br>Alsibai et al. 2023<br>Antunes et al. 2016<br>Avenetti et al. 2021<br>Balakrishnan et al. 2024<br>Campbell et al. 2018<br>Do et al. 2025<br>Dua et al. 2025<br>Hassan et al. 2024<br>Hammadyeh et al. 2019<br>Kapov et al, 2025<br>Khogeer et al. 2025<br>Ma et al. 2024<br>Ramirez et al. 2017<br>Raseena et al. 2020<br>Sawicki et al. 2023<br>Unkel et al. 2022<br>Walsh et al. 2025<br>Wong et al. 2020<br>Yinger et al. 2024<br>Zouaidi et al. 2025
Did not describe parental perceptions	Abdullah et al. 2024<br>Ali et al. 2021<br>Ali et al. 2020<br>Almaeen et al. 2025<br>Brecher et al. 2018<br>Cohen et al. 2022

	Costa et al. 2020
	Davis et al. 2016
	Felemban et al. 2024
	Khubchandani et al. 2022
	Marty et al. 2024
	Marty et al. 2021
	McBeain et al. 2022
	Meghpara et al. 2022
	Nathan 2022
	Nazzal et al. 2021
	Pande et al. 2020
	Randall and Dhar 2023
	Ritwik et al. 2020
	Sakulratchata et al. 2025
	Shindova 2022
	Yost et al. 2019
Sample size < 100	Acharya 2017
	Alanbari et al. 2025
	Ilha et al. 2021
	Ismail et al. 2023
	Rahman et al. 2021
	Malik et al. 2022
	Ramadevi et al. 2024
	Jahanimoghadam et al. 2017
Did not report PPS-specific data	Garret-Bernardin et al. 2017
	Kumar et al. 2024
	Qhreshi et al. 2023
	Segarra et al. 2021
	White et al. 2016

Supplementary Material S2: Full PubMed Search Strategy

Database: PubMed/MEDLINE

Platform: National Library of Medicine

Date of last search: 25 November 2025

(("Behavior Control"[MeSH] OR "Behavior Therapy"[MeSH] OR "behavior management"[tiab] OR "behaviour management"[tiab] OR "behavior guidance"[tiab] OR "protective stabilization"[tiab] OR "protective stabilisation"[tiab] OR "passive restraint"[tiab] OR "physical restraint"[tiab] OR "papoose board"[tiab] OR "pedi-wrap"[tiab])) AND (("Parents"[MeSH] OR parent*[tiab] OR caregiver*[tiab] OR mother*[tiab] OR father*[tiab])) AND (("Dental Care for Children"[MeSH] OR "Pediatric Dentistry"[MeSH] OR "dental pulp"[MeSH] OR "Pulp Therapy"[MeSH] OR "pulp therap*"[tiab] OR "pulp treatment"[tiab] OR "pediatric dental treatment"[tiab] OR "paediatric dental treatment"[tiab])) AND (("Cross-Sectional Studies"[MeSH] OR "cross-sectional"[tiab] OR "survey"[tiab] OR "questionnaire"[tiab]))

Filters Applied:

Language: English

Publication date: January 1, 2015 to November 25, 2025

Humans

No automatic study design filters were applied beyond search terms to avoid missing potentially eligible studies.
